# The differential diagnostic value of serum homocysteine for white coat hypertension

**DOI:** 10.18632/oncotarget.21020

**Published:** 2017-09-18

**Authors:** Shitian Guo, Hui Lin, Sunlei Pan, Xiaoya Zhai, Liping Meng

**Affiliations:** ^1^ School of Medicine, Hangzhou Normal University, Hangzhou, Zhejiang, China; ^2^ Department of Cardiology, Shaoxing People's Hospital, Shaoxing Hospital of Zhejiang University, Shaoxing, Zhejiang, China; ^3^ The First Clinical Medical College, Wenzhou Medical University, Wenzhou, Zhejiang, China

**Keywords:** white coat hypertension, Hcy, diagnosis, hypertension, ABPM

## Abstract

**Objective:**

To assess the value of serum homocysteine (Hcy) in differential diagnosis of white coat hypertension (WCH).

**Results:**

In this retrospective study, serum Hcy levels were elevated in hypertensive patients (*P* < 0.001) compared to WCH patients. Serum Hcy levels were positively correlated with 24-h mean systolic blood pressure, *r* = 0.1378, *P* < 0.001. The results of the receiving operating characteristic (ROC) curve showed that the AUC value of Hcy was 0.80 (95% CI, 0.77–0.83), the cut-off value was 13.8 μmol/L, the sensitivity was 68.58% and the specificity 87.21%. In the prospective study, the AUC value of Hcy was 0.73 (95% CI: 0.67–0.78), higher than N - terminal pro - brain natriuretic peptide(NT-pro-BNP) (0.64, 95% CI:0.58–0.70) and cystatin C (Cys-C) (0.62, 95% CI:0.55–0.68). Hcy, NT-proBNP and Cys-C combined, provided a better indication of a differential diagnosis of WCH, than Hcy alone.

**Materials and Methods:**

This investigation involved both a retrospective and a prospective study. Clinical data including blood pressure, age, sex, height, weight, BMI, smoking status, past history, and behavioral electrocardiogram of patients who had undergone 24-hour ambulatory blood pressure monitoring (ABPM) with elevated clinical blood pressure (BP) were recorded. Pearson correlation analysis was used to test the correlation between Hcy and BP. The ROC curve was used to analyze the value of measuring Hcy levels in differential diagnosis of WCH.

**Conclusions:**

Serum Hcy was decreased in WCH patients and therefore could be a biomarker for differential diagnosis of WCH.

## INTRODUCTION

Hypertension is a modifiable risk factor for cardiovascular events, and anti-hypertensive treatment is the cornerstone for the management [1]. However, it has been recognized increasingly that blood pressure (BP) measured in the clinic room may not truly represent BP levels outside the hospital. This is because the anxiety and nerves elicited by nurses or doctors during clinic room BP measurement may substantially impair the accuracy of this time-honored BP measurement approach in estimating real life BP levels [2].

White coat hypertension (WCH) also referred to as isolated office or isolated clinic hypertension, is used to define patients with elevated clinic BP at repeated visits, but who show normal BP outside the doctor's office. This difference is detected either through ambulatory BP monitoring (ABPM) or home BP monitoring [3]. WCH occurs in 15% to 30% of subjects with an elevated office BP, and the phenomenon is reasonably reproducible [4]. Although there are no pathognomonic diagnostic features of WCH, the condition occurs more frequently in older people, and is more common in women. Other associated factors are, being a non-smoker, pregnancy, having no evidence of target organ damage, and being only recently diagnosed with mild hypertension and then only through a limited number of conventional in-office BP measurements [5].

Previous studies found that WCH patients were at a substantially reduced risk of morbidity compared with sustained mild hypertension patients [6, 7]. The misdiagnosis of subjects with WCH as being truly hypertensive can result in them being penalized in employment and insurance ratings, as well as being prescribed unnecessary lifelong treatment that could potentially have debilitating side effects, especially in the elderly [8]. Moreover, failure to identify the condition results in a large expenditure on unnecessary drugs [9]. For this reason, it is recommended that WCH be ruled out in low risk patients with mild to moderate hypertension, before starting antihypertensive treatment [10].

24-h-ABPM monitors the patient at multiple times a day. It can provide comprehensive data not only about the mean BP, but also its variability, thus enabling the identification of the white coat effect. ABPM also has a stronger prognostic value in relation to cardiovascular outcomes and provides more accurate BP measurements [11]. So, ABPM is recommended by the 2013 ESH/ESC guidelines for facilitating diagnosis of WCH [12]. However, ABPM does have some limitations, such as limited availability, discomfort, and the reluctance of some patients to participate.

Recently, Courand et al. demonstrated the diagnostic value of N - terminal pro - brain natriuretic peptide (NT-proBNP) to rule out WCH in a prospective study that included 26 WCH patients and 1133 sustained hypertension patients [13]. Further, another study conducted by Ma et al. found that serum lncRNA H19 and MALAT1 were increased in subjects with WCH compared to those with normal BP and hypertension. They concluded that serum lncRNA H19 and MALAT1 could be novel non-invasive biomarkers for the diagnosis of WCH [14]. These studies provided a new paradigm for WCH diagnosis. Hcy is a sulfur-containing amino acid. Hyperhomocysteinemia (HHcy) has been shown in previous cohort and genetic studies to be an independent predictor of adverse cardiovascular outcomes [15]. Based on previous studies that serum Hcy levels are increased in hypertensive patients, and our expertise and research into Hcy [16, 17], we explore in this study the value of Hcy for use in the differential diagnosis of WCH from sustained hypertension.

## RESULTS

### Baseline characteristics of patients in the retrospective study

**Figure 1:**
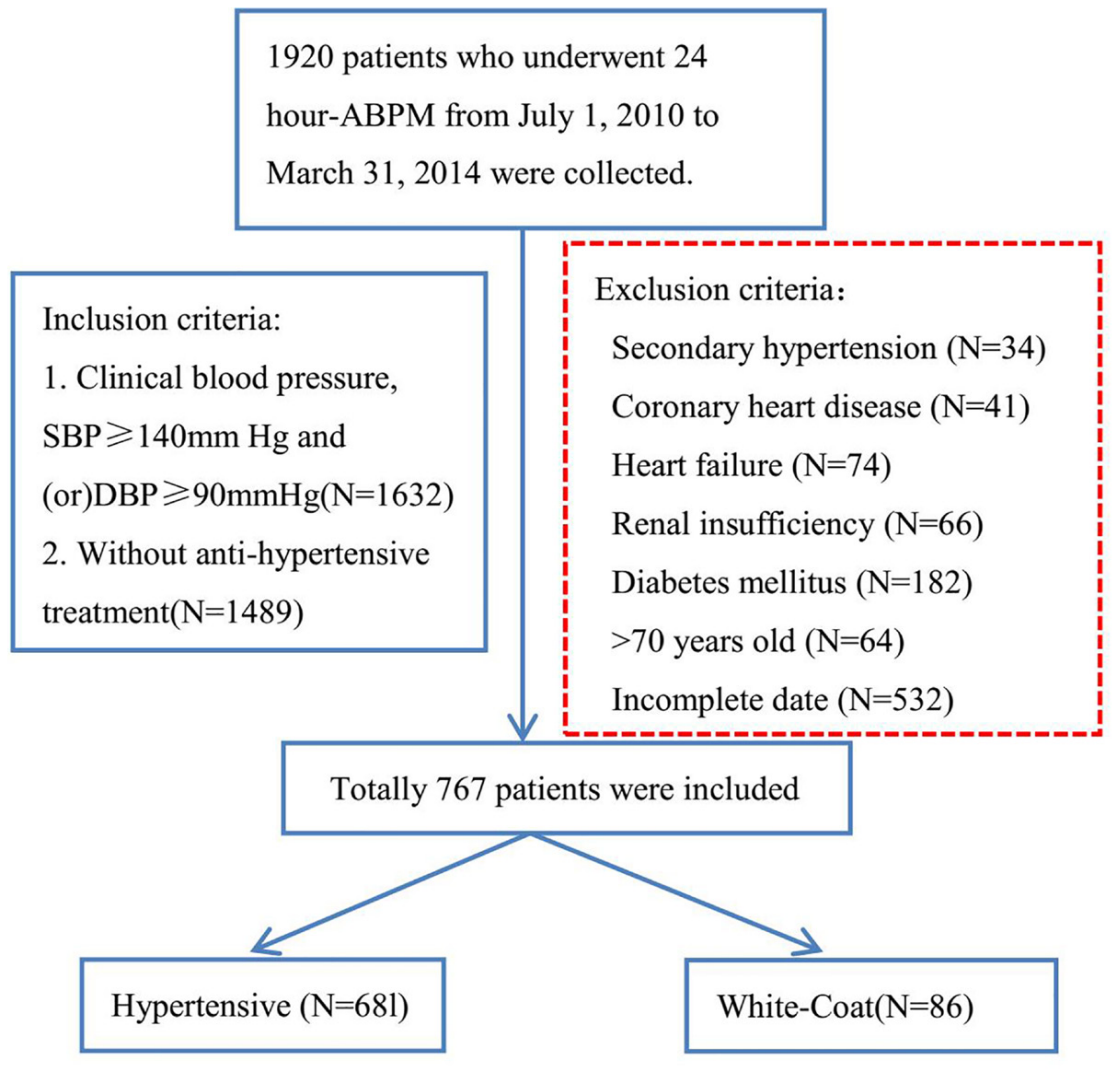
A flow diagram of study participants in the retrospective study ABPM: ambulatory blood pressure monitoring; SBP: systolic blood pressure; DBP: diastolic blood pressure.

Data from 1920 patients who underwent 24-h ABPM were collected. Out of 1920 patients, 767 patients met the inclusion criteria and divided into the WCH group (N=86) and the hypertensive group (*N* = 681) according to the 2013 ESH/ESC guidelines for the management of arterial hypertension [12] (Figure 1).

As shown in Table 1, patients in the hypertensive group were older (58.4 ± 8.6) than patients in the WCH group (54.9 ± 3.2), *P* < 0.001. In the previous study, WCH occurs more frequently in older people [5], our result maybe caused by the excluding of old people who were more than 70 years old. In addition, the alcohol consumption rate was much higher in the hypertensive group. There was no difference in gender, BMI, smoking incidence or family history of hypertension between the two groups. The mean serum Hcy level in the WCH group was 11.1 ± 2.7 μmol/L, significantly lower than that in the hypertensive group. The mean serum levels of HDL, LDL, TG, Cr and eGFR appeared higher in the hypertensive group, but differences were not statistically significant.

**Table 1:** Baseline characteristics of patients of the retrospective study(x ± s)

Characteristics	White-Coat (*N* = 86)	Hypertensive (*N* = 681)	*P* value
Male (%)	49(57.0%)	395 (58.0%)	0.856
Age	54.9 ± 3.2	58.4 ± 8.6	< 0.001^*^
BMI (kg/m2)	20.2 ± 1.4	19.8 ± 2.0	0.631
Smoking (%)	27 (31.4%)	230 (33.7%)	0.660
Alcohol (%)	38 (44.2%)	391 (57.4%)	0.020^*^
Family history of hypertension (%)	11 (13.6%)	143 (21.0%)	0.073
HDL (mg/dL)	53 ± 12.5	59 ± 15.6	0.126
LDL (mg/dL)	102.5 ± 20.8	116.1 ± 21.0	0.063
TG (mg/dL)	181.6 ± 37.1	189.5 ± 29.9	0.072
Cr(μmol/L)	85.1 ± 3.3	87.1 ± 2.8	0.821
eGFR (mL/min)	101.4 ± 8.7	105.1 ± 13.0	0.412
Hcy (μmol/L)	11.1 ± 2.7	16.4 ± 5.4	< 0.001^*^

HDL, High-density lipoprotein; LDL, Low-density lipoprotein; TG, Triglyceride; Cr: creatinine; eGFR, estimated glomerular filtration rate; Hcy, Homocysteine.

^*^P < 0.05.

### BP of patients in the retrospective study

As shown in Table 2, when measured in-clinic, there was no difference in systolic BP (SBP) between the two groups. However, the diastolic BP (DBP) of the WCH group (81.6 ± 7.1) was much lower than the hypertensive group (89.5 ± 9.9), *P* < 0.001. For 24-h BP, regardless of SBP or DBP, all kinds of BP were higher for the hypertensive group than the WCH group (*P* < 0.05 for all).

**Table 2:** Blood pressure of the patients of the retrospective study (x ± s)

	White-Coat (*N* = 86)	Hypertensive(*N* = 681)	*P* value
Clinical blood pressure			
SBP (mm Hg)	149.5 ± 10.8	156.1 ± 11.0	0.063
DBP (mm Hg)	81.6 ± 7.1	89.5 ± 9.9	< 0.001^*^
24-hour BP			
Daytime ( awake)			
SBP (mm Hg)	127.6 ± 12.4	153.7 ± 16.2	< 0.001^*^
DBP (mm Hg)	81.3 ± 11.3	88.8 ± 10.6	< 0.001^*^
Nighttime ( asleep)			
SBP (mm Hg)	113.9 ± 9.4	136.5 ± 13.6	< 0.001^*^
DBP (mm Hg)	63.5 ± 11.7	67.1 ± 8.9	0.041^*^
24-h ABP			
SBP (mm Hg)	121.4 ± 8.7	145.1 ± 13.0	< 0.001^*^
DBP (mm Hg)	71.3 ± 7.1	84.5 ± 8.2	< 0.001^*^

SBP, systolic blood pressure; DBP, diastolic blood pressure; ABP: average blood pressure

^*^P < 0.05.

### Correlation of serum biochemical factors with BP of patients in the retrospective study

**Figure 2:**
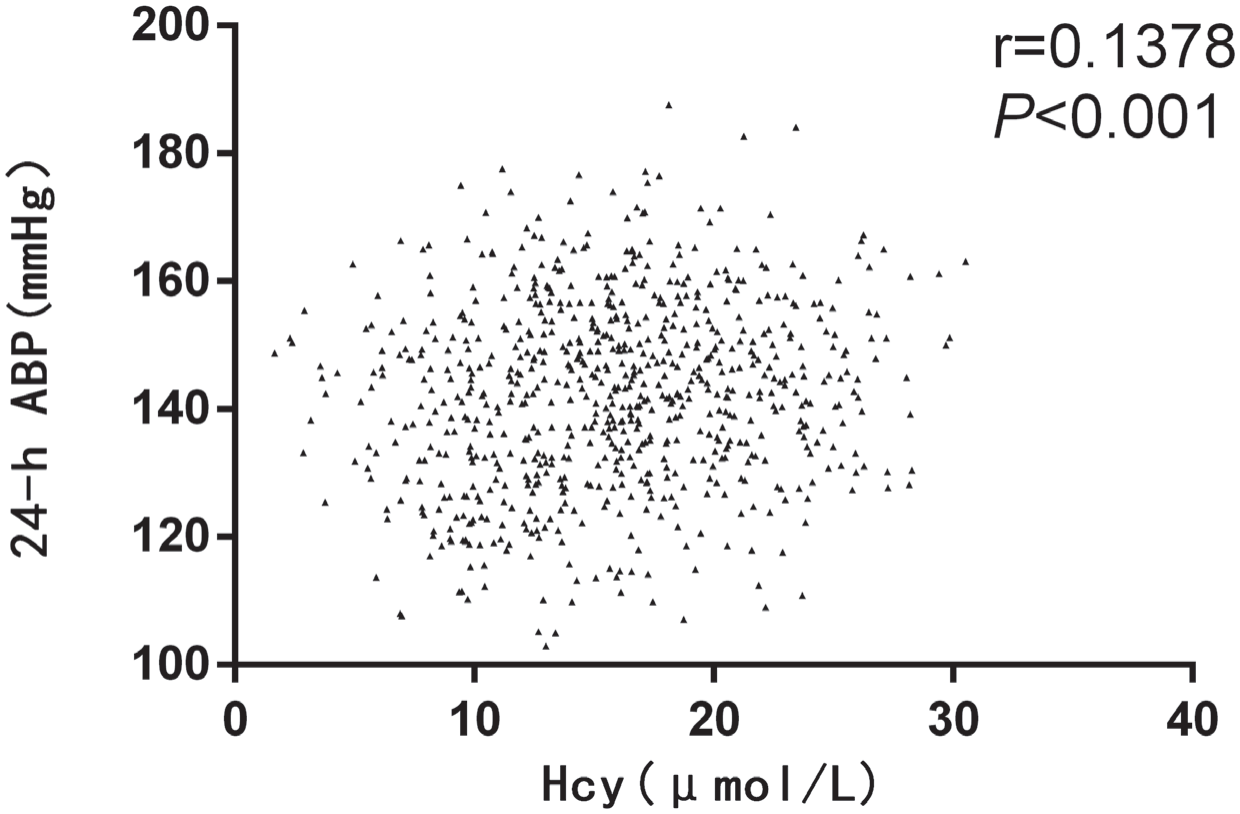
Correlation analysis of homocysteine (Hcy) with blood pressure (BP) in the retrospective study ABP: average blood pressure.

Correlation analyses between levels of serum biochemical factors and BP were performed. The serum Hcy level was positively correlated with the 24-h average BP(ABP) (Figure 2). Other than this, there were no rectilinear correlations between HDL, LDL, TG, Cr or eGFR with BP.

### ROC analysis of serum Hcy as a diagnostic marker of WCH in the retrospective study

**Figure 3:**
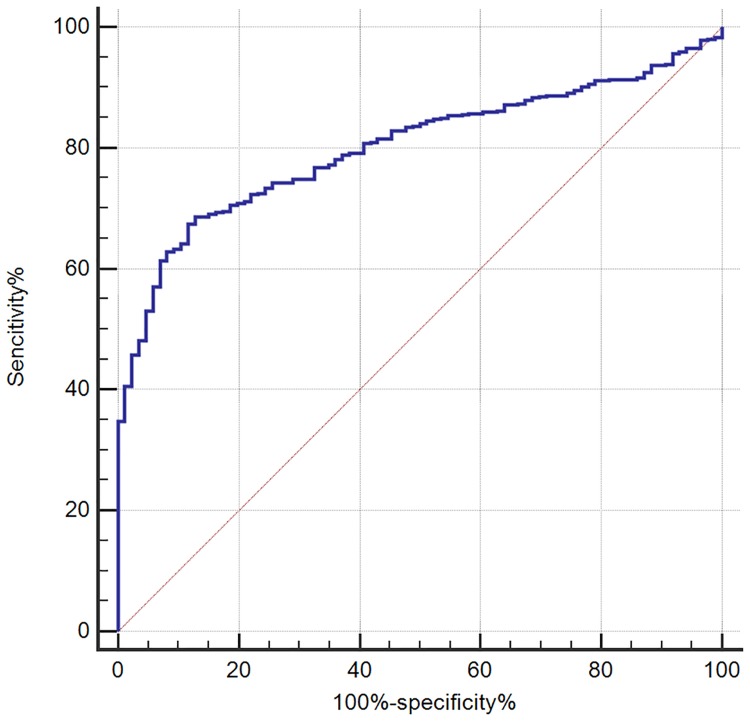
Receiving operating characteristic (ROC) analysis of serum Hcy as a tool for differential diagnosis of white coat hypertension (WCH) in the retrospective study

With a receiver operating characteristic curve (auROC) of 0.80 (95% CI: 0.77–0.83), serum Hcy seems to be a good biomarker for differentially diagnosing WCH from sustained hypertension. When using a best cut-off value of 13.8 μmol/L, its sensitivity was 68.58%, and the specificity was 87.21% (Figure 3 and Table 3).

**Table 3:** ROC analysis of serum Hcy on the diagnosis of White-Coat Hypertension in the retrospective study

	auROC	95% CI	*P*	Youden	Cut-off (μmol/L)	Sensitivity (%)	Specitivity (%)
Hcy	0.80	0.77–0.83	< 0.001^*^	0.56	13.8	68.58	87.21

Hcy, Homocysteine

^*^P < 0.05.

### Baseline characteristics of patients in the prospective study

**Figure 4:**
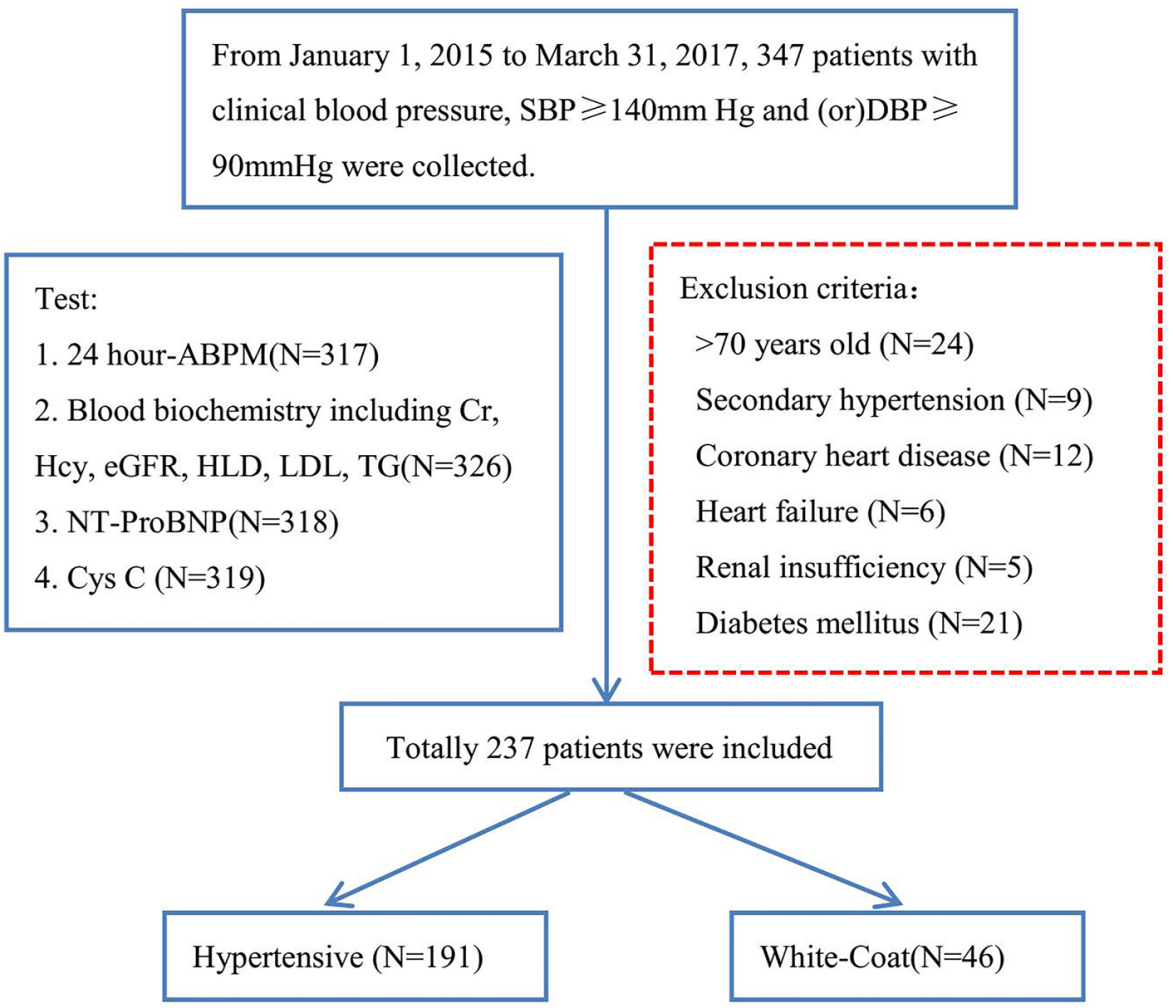
A flow diagram of study participants in the prospective study SBP: systolic blood pressure; DBP: diastolic blood pressure; ABPM: ambulatory blood pressure monitoring; Hcy: Homocysteine; Cr: Creatinin; NT-proBNP: N - terminal pro - brain natriuretic peptide.

From January 1, 2015 to March 31, 2017, data from 347 patients with clinical BP, SBP ≥ 140 mmHg and/or DBP ≥ 90 mmHg were collected. As shown in Figure 4, patients more than 70 years old or with secondary hypertension, coronary heart disease, heart failure, renal insufficiency or diabetes mellitus were excluded. The 24-h ABPM was then used to split the remaining patients into the hypertensive group (*N* = 191) and WCH group (*N* = 46).

The proportion of male patients in the hypertensive group (51.8%) was much higher than that in the WCH group. This may be due to women being more prone to nervousness, and their BP therefore being more affected by entering the clinic. This result was consistent with other research [18, 19]. In addition, hypertensive patients had higher BMI and family history of hypertension than WCH patients. These two indexes have been highlighted as independent risk factors for hypertension [20, 21]. There was no difference in age, smoking incidence or alcohol consumption rate between the two groups. In blood tests, the results were consistent with the retrospective study. The mean serum Hcy levels of the WCH group were lower than that of the hypertensive group. And there were no differences in HDL, LDL, TG, Cr and eGFR levels between the two groups. We also tested the levels of Cys-C and NT-proBNP and found that hypertensive patients had higher levels of Cys-C and NT-proBNP than WCH patients (Table 4).

**Table 4:** Baseline characteristics of patients of the prospective study (x ± s)

Characteristics	White-Coat (*N* = 46)	Hypertensive (*N* = 191)	*P* value
Male (%)	14 (30.4%)	99 (51.8%)	0.009^*^
Age	55.2 ± 5.8	57.9 ± 10.2	0.215
BMI (kg/m^2^)	18.4 ± 2.1	22.1 ± 3.1	0.041^*^
Smoking (%)	15 (32.6%)	56 (29.3%)	0.662
Alcohol (%)	31 (67.4%)	141 (73.8%)	0.380
Family history of hypertension (%)	4 (8.7%)	57 (29.8%)	0.005^*^
HDL (mg/dL)	51.6 ± 7.7	56.1± 11.6	0.281
LDL (mg/dL)	95.3 ± 13.6	124.7 ± 17.3	0.040
TG (mg/dL)	170.4 ± 26.1	190.4 ± 26.3	0.052
Cr (μmol/L)	87.3 ± 4.2	86.2 ± 3.6	0.719
eGFR (mL/min)	100.5 ± 5.8	102.2 ± 9.3	0.508
Hcy (μmol/L)	11.9 ± 3.9	17.3 ± 6.2	< 0.001^*^
Cys-C (mg/L)	1.2 ± 0.3	1.5 ± 0.5	< 0.001^*^
NT-proBNP(pg/ml)	82 ± 17.2	103 ± 23.6	0.038^*^

BMI, Body Mass Index; HDL, High-density lipoprotein; LDL, Low-density lipoprotein; TG, Triglyceride; Cr: creatinine; eGFR, estimated glomerular filtration rate; Hcy, Homocysteine; Cys-C, Cystatin C; NT-proBNP, N - terminal pro - brain natriuretic peptide.

^*^P < 0.05

### BP of patients in the prospective study

As shown in Table 5, the results were comparable with those in the retrospective study. For clinical BP, there was no difference in SBP between the two groups. And the DBP of WCH patients (74.2 ± 8.9) was much lower than observed in hypertensive patients (84.1 ± 13.6), *P* < 0.001. Pure diastolic WCH appears to be exceedingly rare. This result indicated that most WCH presented with high SBP and normal DBP. This could imply that WCH is caused by sympathetic nerves, since the sympathetic nervous system mainly raises SBP [22, 23]. When measured over 24 h, we found that the BP of hypertensive patients was higher than WCH patients except the nighttime DBP (*P* < 0.05).

**Table 5:** Blood pressure of the patients of the prospective study (x ± s)

	White-Coat (*N* = 46)	Hypertensive(*N*= 191)	*P* value
Clinical blood pressure			
SBP (mm Hg)	157.3 ± 9.6	159.4 ± 12.6	0.644
DBP (mm Hg)	74.2 ± 8.9	84.1 ± 13.6	< 0.001^*^
24-hour ABP			
Daytime ( awake)			
SBP (mm Hg)	126.3 ± 10.9	151.5 ± 14.3	< 0.001^*^
DBP (mm Hg)	61.7 ± 9.1	74.8 ± 10.3	< 0.001^*^
Nighttime ( asleep)			
SBP (mm Hg)	94.8 ± 7.3	130 ± 17.2	< 0.001^*^
DBP (mm Hg)	56.4 ± 7.3	62.8 ± 10.4	0.153
24-h			
SBP (mm Hg)	125.7 ± 10.8	146.3 ± 18.4	< 0.001^*^
DBP (mm Hg)	65.3 ± 8.2	70.5 ± 6.4	< 0.001^*^

SBP, systolic blood pressure; DBP, diastolic blood pressure; ABP: average blood pressure

^*^P < 0.05.

### Correlation of serum biochemical factors with BP of patients in the prospective study

**Figure 5:**
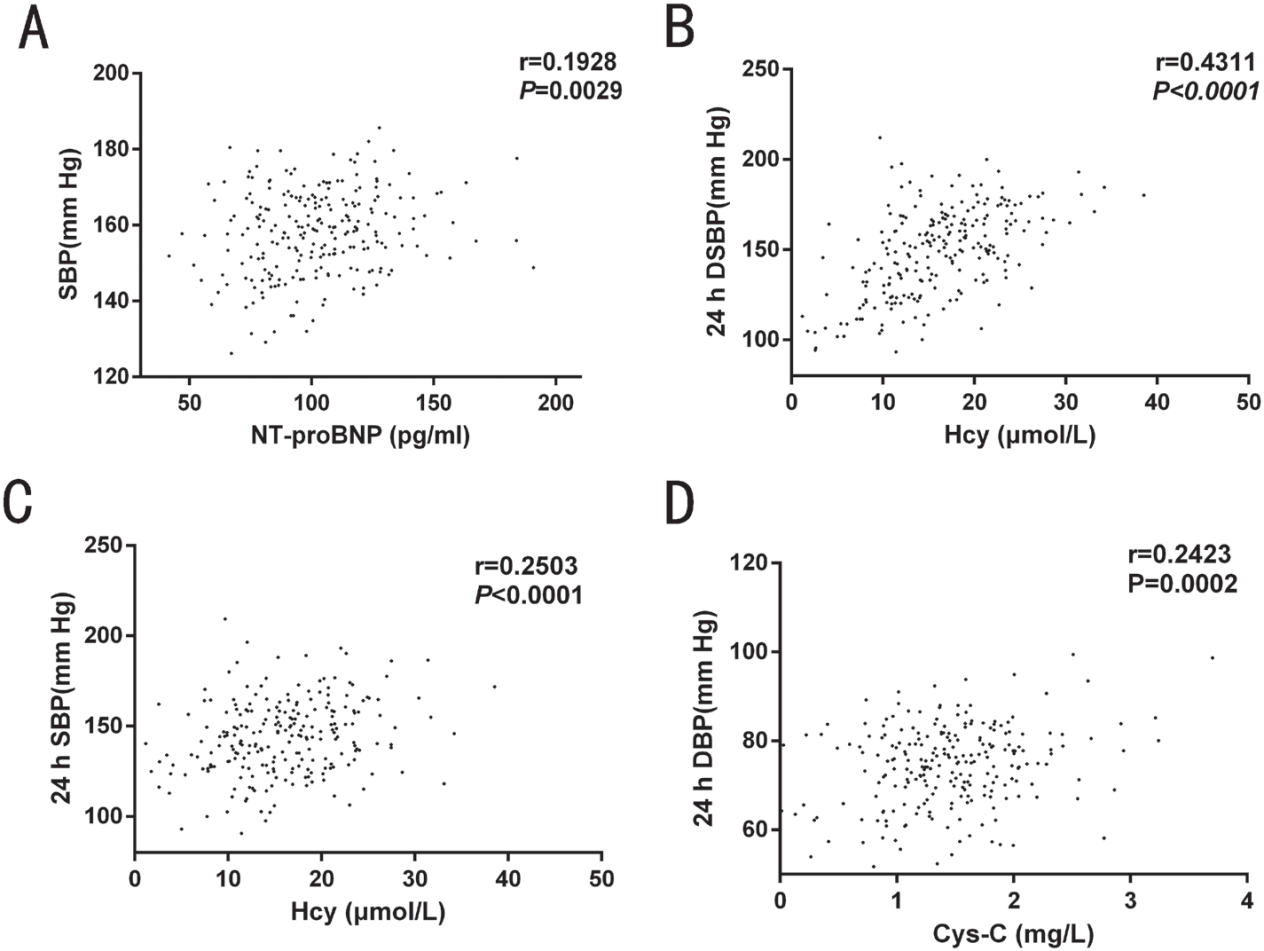
Correlation analysis of serum biochemical factors with blood pressure (BP) in the prospective study (**A)** serum NT-proBNP level was positively correlated with clinical SBP; (**B**–**C**) Serum Hcy level was positively correlated with 24-h daytime SBP and 24-h SBP. (**D**) Cys-C was positively correlated with 24-h average DBP. SPB: systolic blood pressure; DSPB: day-time systolic blood pressure; DBP: diastolic blood pressure.

In the prospective study, we found that serum NT-proBNP levels were positively correlated with clinical SBP (*r* = 0.1928, *P* = 0.0029). This may be because the sudden increase in SBP caused by the white coat effect, affected the heart systolic and diastolic functions, inducing NT-proBNP secretion. Hence, the higher the SBP, the more NT-proBNP was present in the blood. Serum Hcy level was positively correlated with a 24-h daytime SBP (*r* = 0.4311, *P* < 0.0001) and 24-h average SBP (*r* = 0.2503, *P* < 0.0001). In addition, we found that Cys-C was positively correlated with a 24-h average DBP (Figure 5). As signaled by the prospective study, there was no rectilinear correlation between HDL, LDL, TG, Cr or eGFR levels with any BP measurement.

### ROC analysis of serum biochemical factors as diagnostic markers of WCH in the prospective study

**Figure 6:**
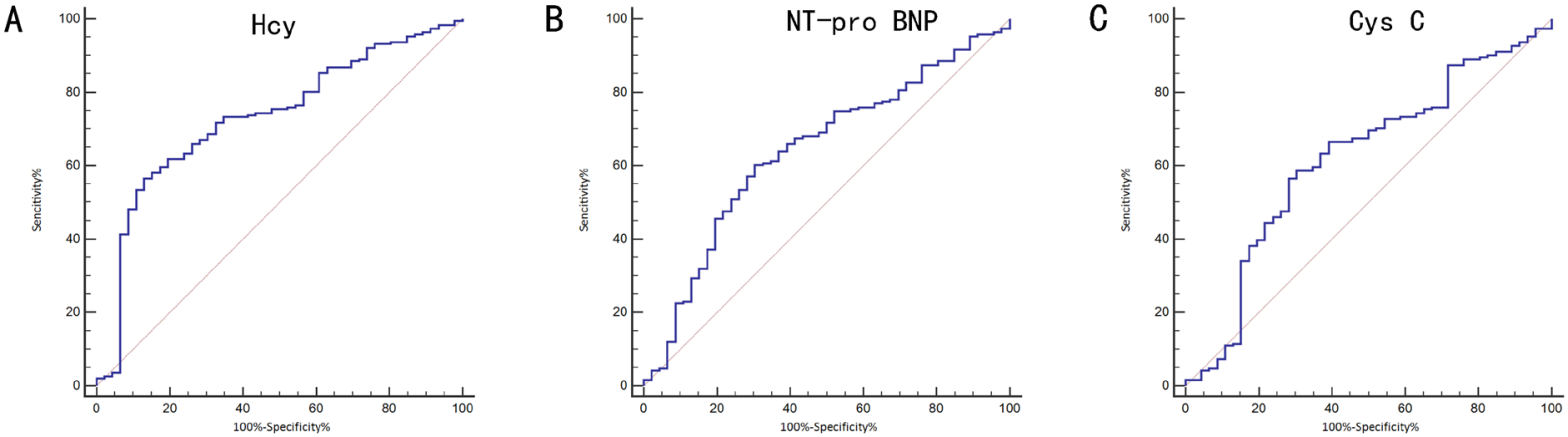
Receiving operating characteristic (ROC) analysis of serum biochemical factors on the differential diagnosis of white coat hypertension (WCH) in the prospective study (**A**) differential diagnosis value of Hcy; (**B**) differential diagnosis value of NT-pro BNP; (**C**) differential diagnosis value of Cys C Hcy: Homocysteine; Cr: Creatinin; NT-proBNP: N - terminal pro - brain natriuretic peptide.

**Figure 7:**
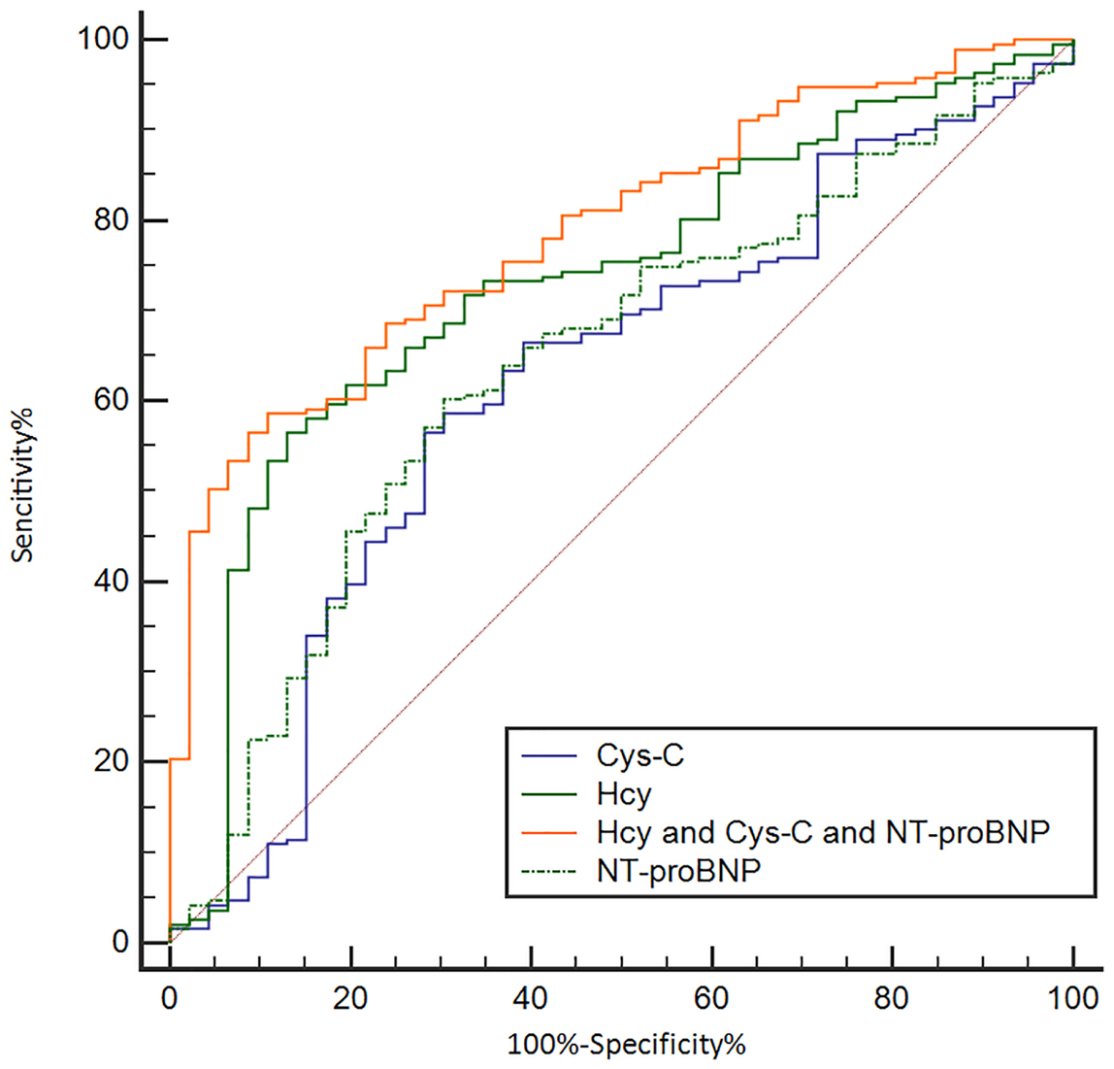
Receiving operating characteristic (ROC) analysis of combined Hcy, NT-proBNP and Cys C as a tool for differentially diagnosing WCH in the prospective study Hcy: Homocysteine; Cr: Creatinin; NT-proBNP: N - terminal pro - brain natriuretic peptide.

Figure 6 and Table 6 show the ability of Hcy, NT-pro BNP and Cys C to differentially diagnose WCH from hypertension. With an auROC value of 0.73 (95% CI: 0.67–0.78), the performance of Hcy was higher than NT-pro-BNP (0.64, 95% CI: 0.58–0.70) and Cys-C (0.62, 95% CI: 0.55–0.68). There was no difference between NTpro-BNP and Cys-C. When using a best cut-off value of 16.45 μmol/L for Hcy, the sensitivity was 56.5% and the specificity was 87.0%. We then combined Hcy, NT-proBNP and Cys C to make a combined Hcy, NT-proBNP and Cys-C score (Hcy & NT-proBNP & Cys-C score = 0.002854+0.24197*-Hcy-0.01385*NT-proBNP -0.51379*Cys-C), of which the coefficients were calculated by multivariate logistic regression with just Hcy, NT-proBNP and Cys-C included. The score had an auROC of 0.79 (95% CI: 0.73–0.84), significantly higher than any of Hcy, NT-proBNP or Cys-C (*P* < 0.05) individually. When we had a cut-off value of 1.82 for this score, the sensitivity was 61.5% and the specificity was 91.3%. Hcy, NT-proBNP and Cys-C combined gave a better performance than Hcy alone (Figure 7).

**Table 6:** ROC analysis of serum Hcy, NT-proBNP and Cys C on the diagnosis of White-Coat Hypertension in the prospective study (x ± s)

	auROC	95% CI	*P*	Youden	Cut-off	Sensitivity(%)	Specitivity(%)
Hcy	0.73	0.67–0.78	< 0.001^*^	0.45	16.45	56.5	87.0
NT-proBNP	0.64	0.58–0.70	0.002^*^	0.30	97.91	60.2	69.6
Cys-C	0.62	0.55–0.68	0.012^*^	0.28	1.44	56.5	71.7
Hcy & NT-proBNP & Cys-C	0.79	0.73–0.84	< 0.001^*^	0.48	1.82	61.5	91.3

Cr: creatinine; Hcy, Homocysteine; Cys-C, Cystatin C; NT-proBNP, N - terminal pro - brain natriuretic peptide.

Hcy & NT-proBNP & Cys-C score = 0.002854+0.24197*Hcy-0.01385*NT-proBNP -0.51379*Cys-C

^*^P < 0.05.

## DISCUSSION

WCH have been reported by clinical studies that accounts for about 4–30% of individuals attending the clinic with elevated BP. The Spanish ABPM Registry recently conducted a study aimed at investigating the prevalence of hypertension phenotypes, showed that WCH represented 24% of cases [24]. Furthermore, the ARTEMIS project which was conducted in five different continents, demonstrated that among 5523 untreated patients with elevated clinic BP, WCH prevalence was approximately 23% [25]. In the PAMELA study, 1657 untreated participants with elevated clinic BP were included, and WCH prevalence ranged from 9 to 12%. In our study, we included patients with elevated clinic BP and divided the participants into a WCH group and sustained hypertension group using 24-h ABPM. In the retrospective study, 11.2% patients were diagnosed as having WCH and in the prospective study the WCH incidence rate was 19.4%. The rate of WCH diagnosis varies greatly in different studies, and this could be due to differing definitions of WCH. In this study, according to the 2013 ESH position paper, we defined WCH as office BP above 140/90 mmHg and mean 24-h BP below 130/80 mmHg [12].

Previous studies found that WCH was less common in thin man, and more common in obese women. Consistent with these studies, our investigation demonstrated that compared to sustained hypertension patients, those with WCH were more likely to be female and more obese. As stated by Bloomfield, WCH is a permanently conditioned reflex from anticipation and fear that BP measurement may indicate future illness [9]. This could explain why WCH is more common in women. Due to its association with obesity and high levels of serum lipids, WCH is considered to be a metabolic syndrome by some researchers [26, 27].

Whether WCH is a benign phenomenon is still under debate. Prospective longitudinal studies have examined the relationship between WCH and cardiovascular risks but delivered inconsistent results [28–30]. Mancia's study demonstrated that, compared with normotensive subjects, the risk of cardiovascular mortality, adjusted for potential confounders, showed a progressive and significant increase in WCH and sustained hypertensive subjects [28]. In addition, Sung's study, that followed 1257 untreated volunteer subjects for 15 years, suggested that arterial aging was the main causative factor of the white coat effect [29]. For this reason, WCH carries higher risk for cardiovascular mortality than normotensives; this is probably a function of the enhanced wave reflections that accompany arterial aging [29]. However, there have also been studies showing that the prognosis of patients with WCH is similar to that of true normotensives [30]. Asayama et al. reported that the risks of cardiovascular disease (CVD) were not increased in patients with WCH considering 24-h ABPM [31]. Additionally, Franklin et al. found that in untreated patients, those with WCH defined by daytime ABPM and patients with normal BP were at similar risk of CVD [8]. The inconsistency between these studies may be caused by: different sample populations at baseline (untreated, treated, or mixed); difference in out-of-office BP monitoring protocol and cut-off values; difference in study characteristics such as endpoint assessment, sample size, and duration of follow up.

Similar to the studies regarding causation, prevalence and prognosis, the appropriate treatment for WCH is also debated. Whether patients with WCH would benefit from antihypertensive treatment remains unknown. The traditional view was that, for WCH patients, the primary aim should be to reduce the patient's worry, not start treatment on a lifetime of unnecessary medication with potential side effects. This would diminish the unnecessary time spent by the physician. Avoiding unnecessary pharmacological treatment is particularly important since it has been shown that antihypertensive treatment for WCH patients might only lower clinic BP, rather than that shown by ABPM [32]. Furthermore, a post-hoc analysis of a subgroup of patients from the Systolic Hypertension in Europe trial showed that in WCH, antihypertensive treatment did not lower the risk of cardiovascular events [33]. Despite these, recent studies have suggested that WCH is not a benign condition. Because WCH is always highly associated with metabolic changes and causes asymptomatic organ damage. In addition, although the risk of cardiovascular events of WCH is lower than sustained hypertension, it is much greater than truly normotensive individuals. A subsequent 2009 PAMELA report did stratify by treatment status and showed that untreated subjects with WCH more frequently developed sustained hypertension, suggesting the potential for increased long-term risk [34]. According to these controversial results, the 2013 ESH/ESC guidelines suggested that with high or very high risk WCH patients, antihypertensive treatment should be given [12].

Regardless of whether antihypertensive treatment is prescribed for WCH, it's necessary to differentiate WCH from sustained hypertension. ABPM is the traditional tool for this process. According to UK guidelines, all patients with stage 1 and 2 hypertension should have ABPM to confirm the diagnosis of hypertension before taking treatment decisions [35]. And according to “The Task Force of the Eighth International Consensus Conference on Blood Pressure Monitoring”, ABPM should be applied to exclude WCH in untreated patients when the office BP is ≥140/90 mmHg [36]. And the NICE guidelines advocate that every person with elevated clinic BP undergo ABPM to rule out WCH for avoiding unnecessary treatment with antihypertensive drugs [37]. However, ABPM does have its limitations, for example it is uncomfortable, with patients often reluctant to participate. In addition, some studies have found that 24-h BP tested by ABPM can vary greatly in the same person. This has resulted in ABPM-confirmed WCH cases which were later shown to be truly sustained hypertension by an ABPM test conducted days later. As such, the European Society of Hypertension Working Group on Blood Pressure Monitoring recommends that once ABPM has confirmed the diagnosis of WCH, it should be reconfirmed in 3 to 6 months by another ABPM test [38, 39].

Recent studies have provided a new tool for WCH diagnosis. Courand et al. demonstrated the diagnostic value of NT-proBNP to separate WCH from hypertension patients [13]. In their study, 1159 patients were divided into 26 WCH patients and 1133 sustained hypertension patients. They found that NT-proBNP was significantly lower in patients with WCH and the ROC curve of plasma NT-proBNP to differentiate WCH from sustained hypertension had an AUC at 0.662 with sensitivity 44.0% and specificity 96.1% [13]. Consistent with this study, we also found that NT-proBNP was lower in WCH patients. In addition, the AUC value of NT-proBNP for differentiating WCH from sustained hypertension was 0.64, with sensitivity 60.2.0% and specificity 69.6%. All these results indicate the diagnostic value of NT-proBNP for separating WCH from hypertension patients. A study conducted by Ma et al. found that serum lncRNA H19 and MALAT1 were increased in subjects with WCH compared to those with normal BP or hypertension. They concluded that serum lncRNA H19 and MALAT1 could be novel, non-invasive biomarkers for the diagnosis of WCH [14]. In our study, we found that serum Hcy, with an auROC of 0.80 (95% CI: 0.77–0.83), could also be a good biomarker for the differential diagnosis of WCH.

Some studies indicate that HHcy could play a role in the development of elevated BP [40, 41]. In our previous studies, we highlighted the ability of Hcy to induce vascular smooth muscle cell migration and proliferation [42], promoting vascular remodeling [43] and inhibiting endothelial cell proliferation [44]. These could be possible mechanisms by which Hcy causes vascular damage and eventually, hypertension. These previous studies demonstrated that Hcy was involved in the development of hypertension. In our study, we found Hcy levels were lower in WCH, as compared with hypertension patients. This result was consisted with the study conducted at 2003 by Pierdomenico et al. which demonstrated that middle-aged WCH patients had lower circulating Hcy levels than sustained hypertensive patients [45]. Our study also revealed that Cys-C had differential diagnostic value for identifying WCH. With this in mind, we combined Hcy, NT-proBNP and Cys C to make a Hcy, NT-proBNP and Cys-C combined score (Hcy & NT-proBNP & Cys-C score = 0.002854+0.24197*Hcy-0.01385*NT-proBNP -0.51379*Cys-C), and found the combination with auROC of 0.79, had a better performance than Hcy alone. Although the AUC value of these serum markers is not high enough and we cannot now make decisions only based on these results without ABPM. In the future, with the ever-increasing advancements in technology and equipment, it will be much simpler to detect several serum biochemical factors in one sample. We believe that combining the detection of Hcy, NT-proBNP, Cys C and other serum biochemical factors could further improve the differential diagnostic value of identifying WCH without ABPM.

In conclusion, we found that compared with the hypertension patients, serum Hcy was decreased in WCH patients. As such, serum Hcy could be a biomarker for the differential diagnosis of WCH. Furthermore, we found that detecting Hcy, NT-proBNP and Cys C together could even further improve the outcomes for differential diagnosis of WCH by serum biochemical factors. This study provides novel possibilities beyond ABPM for the diagnosis of WCH.

## MATERIALS AND METHODS

### Patients

This investigation consisted of a retrospective study and a prospective study. The retrospective study explored the differential diagnostic possibilities of Hcy for WCH, and the prospective study was proposed in order to validate those possibilities.

The retrospective study sample population was collected from July 1, 2010 to March 31, 2014 according to strict inclusion and exclusion criteria. The inclusion criteria were as follows: first, patients must undergo 24-h ABPM at the department of dynamic blood pressure monitoring. Second, the clinical BP of the patients must be SBP ≥ 140 mmHg and/or DBP ≥ 90 mmHg. Third, none of the patients could have received anti-hypertension therapy. Patients more than 70 years old or with a history of coronary heart disease, heart failure, renal insufficiency or diabetes mellitus or those who had not been given the serum Hcy level test were excluded. After these criteria were applied, 767 patients were included. According to 2013 ESH/ESC Guidelines for the management of arterial hypertension [12], these 767 patients were divided into the hypertensive group (24 h ABP ≥ 130/80 mmHg or DBP ≥ 135/85 mmHg, or NBP ≥ 120/70 mmHg, *N* = 681) and the WCH group (clinical SBP ≥ 140/90 mmHg and 24 h ABP <130/80 mmHg and DBP < 135/85 mmHg and NBP <120/70 mmHg, N=86).

The prospective study started January 1, 2015 and ran until March 31, 2017. The inclusion and exclusion criteria were the same as the retrospective study. 237 patients were included and then divided into the hypertensive group (*N* = 191) and the WCH group (*N* = 46).

### Data collection

In the retrospective study, all data were collected from medical records, including BP, age, sex, height, weight, BMI, smoking status, disease history, and biochemical test results.

In the prospective study, clinical BP was tested at least three times using a Riva-Rocci sphygmomanometer with the patient sitting in a quiet environment. A medical history and health habit inventory were taken by a specific doctor. These included demographic characteristics (age, sex, height, weight), traditional cardiovascular risk factors (body mass index, smoking, drinking status (current versus past or never)) and existence or history of any clinical disease (diabetes, previous myocardial infarction, heart failure, renal insufficiency or cancer, this was then validated by a combination of self-report of physician diagnosis and review of medical records).

ABPM was performed using a non-invasive automated device with the cuff fitted on the non-dominant arm, as described in previous studies [46]. The ABPM was only conducted once in this study. BP and heart rate were recorded every 15 min during the day and every 30 min during the night. For the present analyses, we defined daytime and nighttime according to the habitual waking and sleeping patterns reported by each volunteer. To avoid any interference in the ABPM analysis, a report of the normal daily activity of each participant was made. Finally, 24-h ABP, DBP, and NBP were calculated and recorded.

Blood samples were obtained from each subject and then used for biochemical analysis. Centralized analysis of NT-proBNP and creatinine was performed using blood samples obtained at admission. Plasma concentration of NT-proBNP was measured by microparticle enzyme immunoassay. Serum creatinine, Cys C and Hcy was analyzed using commercially available kits from R&D Systems company.

### Statistical analysis

Continuous variables were summarized as mean ± standard deviation (SD), and categorical variables were displayed as counts or percentages. Student-t test was used for continuous variables and χ^2^-test for categorical variables. Associations between serum biochemical factors and BP levels were tested using the Pearson correlation. To assess the diagnostic efficiency of the serum biochemical factors, the area under the receiver operating characteristic curve (auROC) was calculated, since this is a measure of discrimination. Furthermore, the standard indexes of validity, such as the Youden index, sensitivity and specificity were calculated according to ROC results.
